# Bioactivity-explorer: a web application for interactive visualization and exploration of bioactivity data

**DOI:** 10.1186/s13321-019-0370-7

**Published:** 2019-07-10

**Authors:** Lu Liang, Chunfeng Ma, Tengfei Du, Yufei Zhao, Xiaoyong Zhao, Mengmeng Liu, Zhonghua Wang, Jianping Lin

**Affiliations:** 1 State Key Laboratory of Medicinal Chemical Biology, College of Pharmacy and Tianjin Key Laboratory of Molecular Drug Research, Nankai University, Haihe Education Park, 38 Tongyan Road, Tianjin, 300353 China; 2 Tianjin Institute of Industrial Biotechnology, Biodesign Center, Chinese Academy of Sciences, Tianjin, China; 3 Platform of Pharmaceutical Intelligence, Tianjin International Joint Academy of Biomedicine, Tianjin, 300000 China

**Keywords:** Network pharmacology, Web server, Matched molecular pair, Molecule scaffold, ChEMBL

## Abstract

To better leverage the accumulated bioactivity data in the ChEMBL database, we have developed Bioactivity-explorer, a web application for interactive visualization and exploration of the large-scale bioactivity data in ChEMBL. Mining and integration of the Therapeutic Target Database disease-target mapping into the ChEMBL database has enabled Bioactivity-explorer to include 493,430 scaffolds, 31,400,000 matched molecular pairs, 1330,220 target–target interactions in terms of shared active compounds, 4526,718 target–target interactions in terms of shared active scaffolds, 97,041,700 molecule–molecule interactions and 14,974 disease-target mappings. This web tool is available at http://cadd.pharmacy.nankai.edu.cn/b17r. The source codes of the front end and back end, released under MIT license, can be found at GitHub.

**Electronic supplementary material:**

The online version of this article (10.1186/s13321-019-0370-7) contains supplementary material, which is available to authorized users.

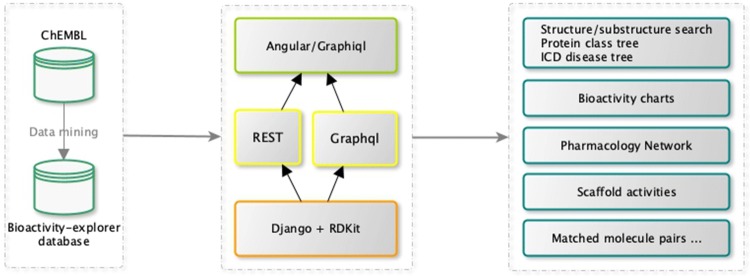

## Introduction

In the past decade, open bioactivity data accumulated rapidly in public data repositories such as ChEMBL [1], BindingDB [2] and PubChem BioAssay [3]. For example, the latest release of the ChEMBL database (v24) contains 12,091 targets, 1828,820 distinct compounds and 15,207,914 activities. With the plethora of publicly available bioactivity data, it becomes increasingly important to extract and explore information of interest in drug discovery, especially information derived from large-scale SAR data, for example, matched molecular pairs (MMPs), which describe how structural transformations affect compound properties, including biological activity [4]; the scaffold distribution of a specific target [5]; and network pharmacology [6], which relates targets in terms of shared active ligands, etc. Many studies have been performed to mine the information underlying experimental data repositories. Ye et al. explored all the scaffolds of compounds from the ChEMBL database [5, 7]. Paolini et al. [8] presented a global mapping of pharmacological space to explore the global relationships between chemical structure and biological targets. By employing the similarity ensemble approach, Keiser et al. related protein pharmacology to ligand chemistry, which was found to reveal unexpected protein target relationships that may be assayed using the ligands themselves. Wirth et al. [9] built an MMP database, named SwissBioisostere, by mining the bioactivity data in ChEMBL. Weber et al. also reported an MMP database with 3D information obtained by mining the data from ChEMBL and PDBbind.

However, to our knowledge, there is no tool or web server that provides comprehensive information mining from public bioactivity repositories, as mentioned above. Among the public bioactivity data repositories, the ChEMBL database hosted by the European Bioinformatics Institute is one of the most widely used data sources because of its well-curated bioactivity data and user-friendly downloading format [1]. Accordingly, we have developed Bioactivity-explorer, a web application for the interactive visualization and exploration of large-scale bioactivity data in the ChEMBL database.

## Implementation

### Data preparation

The ChEMBL database is the central data repository for Bioactivity-explorer. To maintain consistency with the release pace of the ChEMBL database, we have developed an in-house workflow to help updating data which will be released on GitHub page. Current data were retrieved from the latest ChEMBL (v24) together with the target-diseases data (the ICD-10 diseases classification) from the TTD database [10], in which diseases are mapped to ChEMBL targets through Uniprot ID. For example, there are three targets, “Maltase-glucoamylase, intestinal”, “Fibrin” and “Recombinant tetravalent attenuated live dengue”, associated with Dengue fever(A90), and of them the target “Maltase-glucoamylase, intestinal” has Uniprot ID “O43451” which was then mapped to Maltase-glucoamylase (CHEMBL2074) in ChEMBL. To mine more information, essentially following our previous work [11], the bioactivity data from ChEMBL were first prepared by the following steps:

1. Only activities with valid pChEMBL values (negative logarithms of molar IC50, EC50, Ki, etc.) were considered for future usage, such as scaffold activity, target–target network and MMPs generation, and for molecules with multiple potency measurements reported, the aggregate values (including mean, median, maximum and minimum value) were calculated.
2. The Bemis-Murcko (BM) method [12], which is one of the most widely used scaffold-generation methods, was used to generate scaffolds for all structures. The algorithm implemented in RDKit was employed.
3. As in the curation procedure for molecule activity, the aggregated BM scaffold [12] activity values were generated from molecules of the scaffold.
4. The definition of “interaction” of target–target and molecule–molecule were derived from the network pharmacology work by Paolini et al. [8]. Similar ideas were successfully used in the visualization of the chemical space [13] and target prediction [14]. Hu et al. [15] employ a simple definition that two targets are related to each other if they share at least five active compounds. Inspired by the above studies, the target–target and molecule–molecule interaction relationships, which were defined by the number of shared active molecules/targets, were pre-calculated based on the processed molecule/scaffold activities mentioned above. For example, in the current database there are 57 and 143 ligands with valid pChEMBL against Sucrase-isomaltase (CHEMBL2748) and Maltase-glucoamylase (CHEMBL2074), respectively, and 51 of them are in common. This aggregated data was store in database for target–target network view. It should be mentioned that, in the molecule–molecule interaction generation process, for simplicity, only small molecules with more than 10 activities reported were considered, and only molecular pairs that shared more than 5 targets were stored in the database.

For MMP identification of compounds from the same assay, an algorithm implemented in RDKit [16] was employed, and of the generated transformations, the one with most heavy atoms (to maximize the transformation) was selected.

### Construction

A user-friendly web interface was developed by combining the Angular framework (https://angular.io) and the Django framework (https://www.djangoproject.com/). All data used were stored in a PostgreSQL database. We also implemented a graphql interface (https://graphql.org/), which offers greater query flexibility for the user; a use case is provided in the Results section. The docker files for both the front end and the back end of the tool are provided to facilitate developing, deploying and running the application. Detailed setup instructions can be found at the GitHub repository.

## Results

The front end of Bioactivity-explorer was built with modern web technology, Angular (www.angular.io) and Angular Material; therefore, to avoid compatibility problems, we strongly recommend the use of the latest versions of web browsers (Google Chrome, Firefox, etc.). Bioactivity-explorer consists of the following main modules: a target/molecule search panel, a target page, a molecule page, a scaffold page, an assay page and a document page.

### Data search and results table

**Fig. 1:**
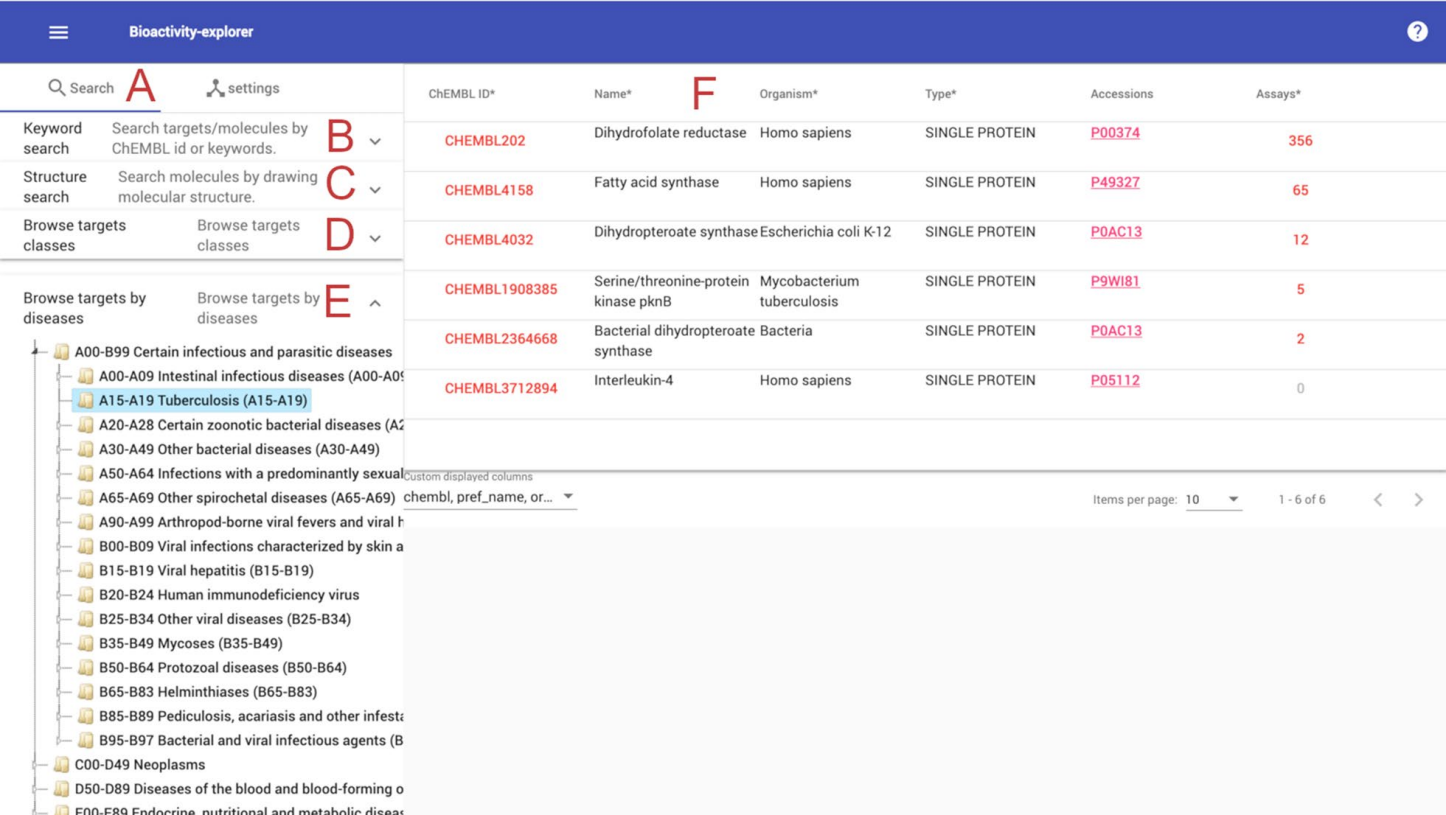
Browsing the target activities of Tuberculosis (A15–A19). Users can perform a keyword search (B), perform a structure search (C), or browse targets by target classification (D) or ICD disease classification (E). Click the menu icon (A) to show or hide the search panel. The table in the right (F) is the result activities. Click ChEMBL ID, accession code and assay number to view corresponding page. Columns followed by “*” are sortable

As shown in Fig. 1, Bioactivity-explorer provides a target/molecule keyword search, molecular structure search, and browser targets by classification. Bioactivity-explorer provides two kinds of target classification: (1) the protein classification taken from the official ChEMBL database and (2) the protein-disease classification derived from the latest Therapeutic Target Database (TTD) [10] by mapping targets to the UniProt accession id. The keyword search supports the ChEMBL ID, target and molecule name as input. For the structure search, the JSME applet [17] was employed for drawing structures, and then structure and substructure searches of molecules or scaffolds can be performed. The Morgan fingerprint (implemented in RDKit) similarity threshold is adjustable from 0.7 to 1, and set to 1 enables an exact structure search.

### Target information page

**Fig. 2:**
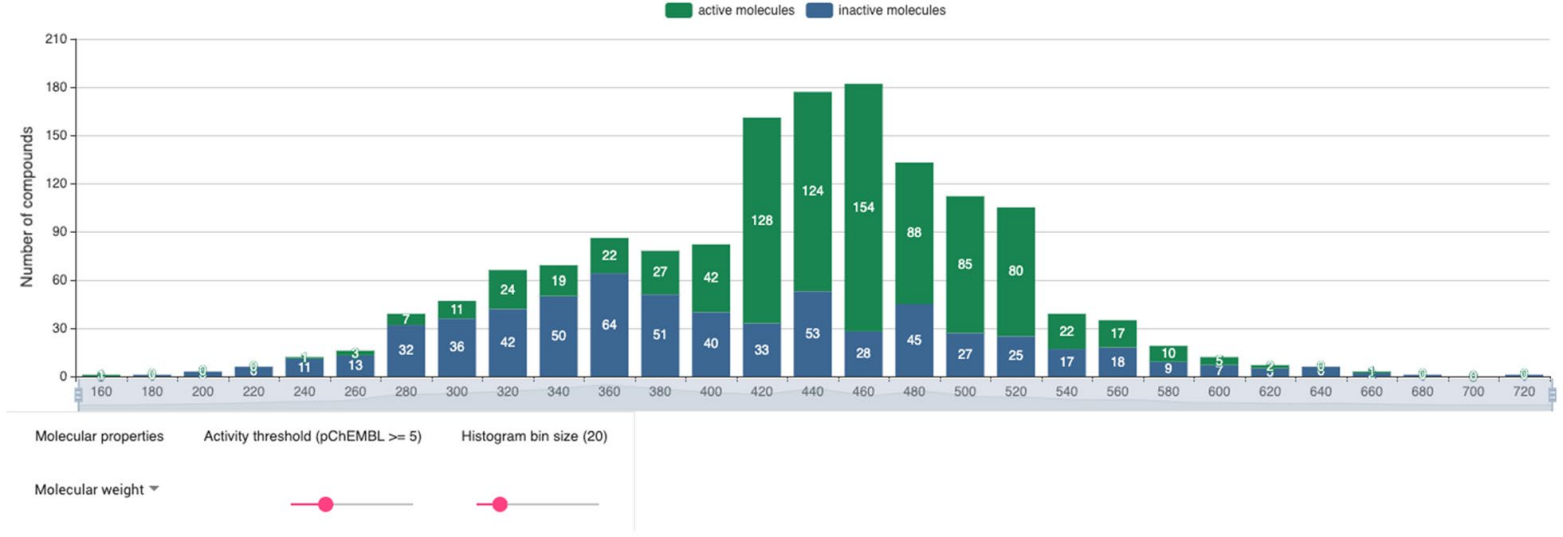
Target-associated compound property histogram. Active and inactive molecules are shown in different colors. Below the histogram, the molecule property list, activity threshold and bin size threshold slider options could be used to interact with the chart

One of the central features of Bioactivity-explorer is the target information page. In addition to the basic information on a target, to improve usability, bioactivities, charts based on bioactivity data, target interaction network, MMPs and active scaffolds are also organized into this page. Compared to the charts of a target at the official ChEMBL site, Bioactivity-explorer provides charts with more functionalities. Taking the ligand property histogram as an example (Fig. 2), the chart in Bioactivity-explorer was further associated with bioactivities. Active and inactive molecules were shown in different colors; therefore, by selecting different activity thresholds and bin sizes, the user can view the compound properties interactively, including the molecular weight, polar surface area (PSA), AlogP, number of heavy atoms, number of aromatic rings, number of rotatable bonds (RTB), number of hydrogen bond donors (HBD) and number of hydrogen bond acceptors (HBA). There is an additional chart about the number of papers published each year on the target, which gives an overview of the research trend of this target.

**Fig. 3:**
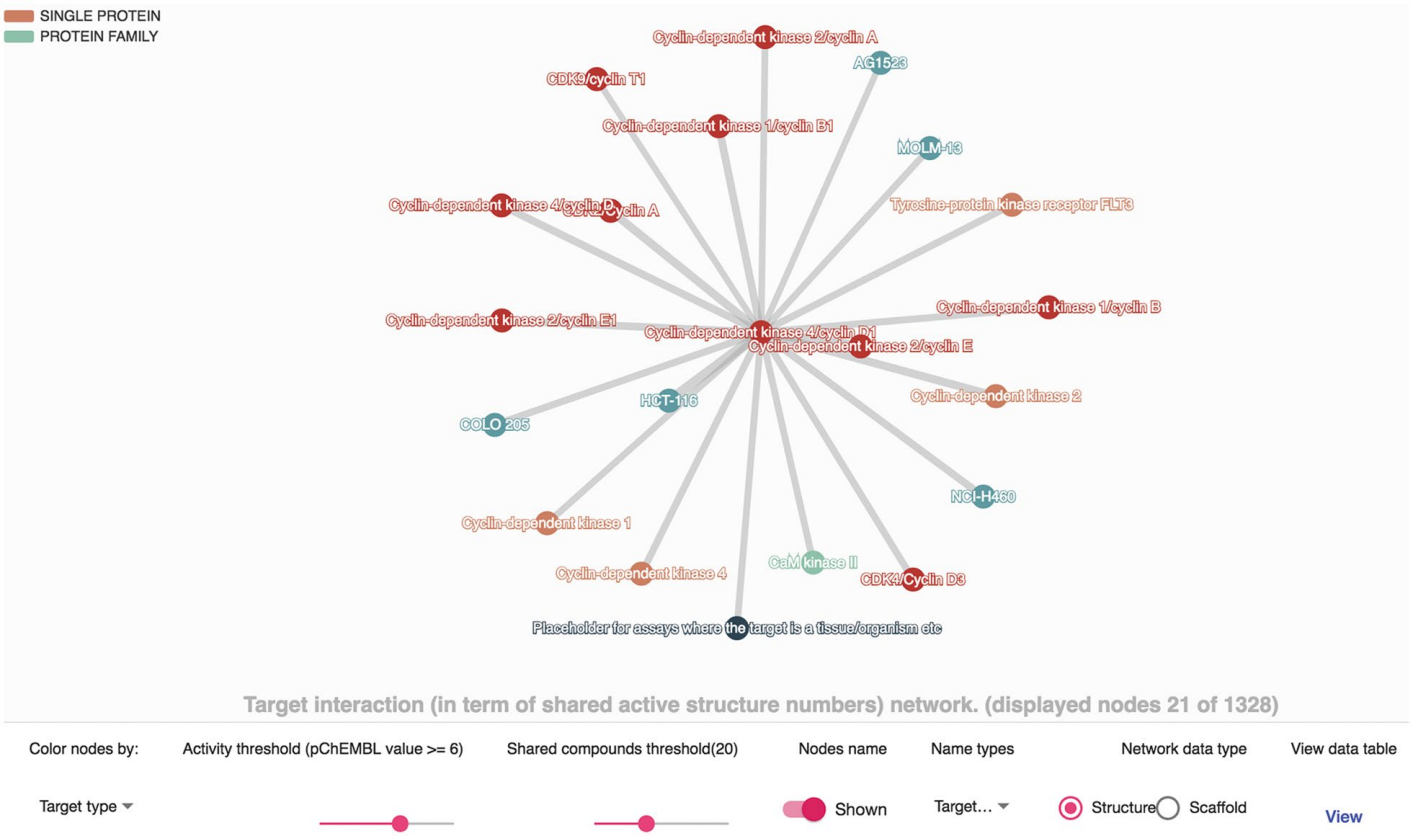
Target interaction network of Cyclin-dependent kinase 4/cyclin D1. The node and edge denote the target and target–target interaction relationship, and double click on them to view target information and the shared compounds list, respectively. The options below were used to interact with the network

As stated above, the target interaction relationship here was defined by the number of shared active compounds or scaffolds. The “Network” tab on the target page contains a network, as shown in Fig. 3, illustrating these relationships. An edge that connects two nodes in the network denotes that the two targets have active compounds in common, and the width is proportional to the number of shared compounds. To construct an interactive network view, the node color (color by target type or organism), network type (shared compounds or scaffolds), activity threshold and number of shared compound thresholds are also customizable. The node and edge can be double-clicked to view the corresponding target and compound list, respectively. For some targets with a plethora of bioactivity data, it may take seconds to load the network.

**Fig. 4:**
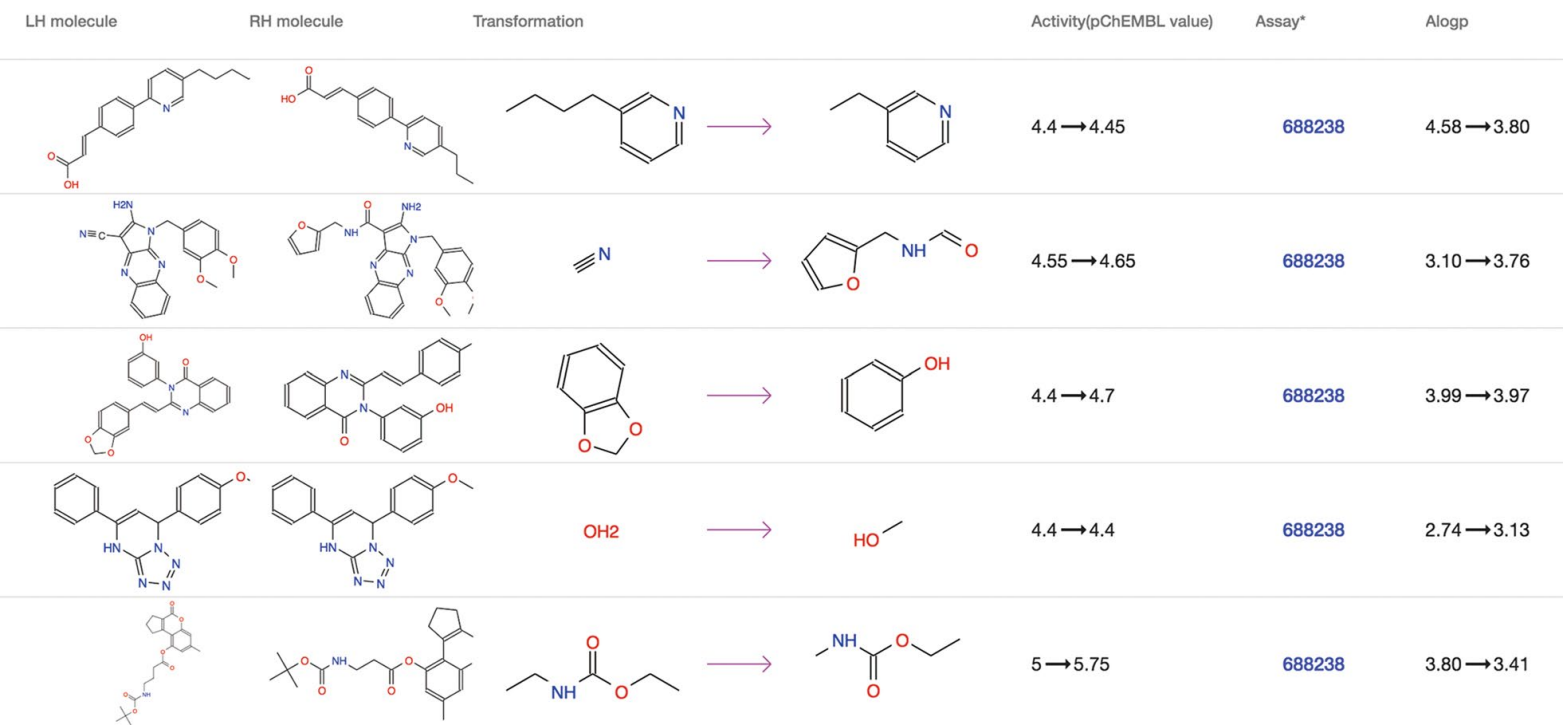
An example of the MMP table. Each row of the table represents an MMP, including the left handed (LH) and right handed (RH) molecule, structural transformation, activity and property changes

The “MMP” tab on the target page contains a table of all MMPs derived from the assays of the target (Fig. 4). Each row of the MMP table is a matched molecular pair and consists of a right handed and left handed molecular structure, structural transformation, activity change, assays and physical property changes including molecular weight, PSA, RTB and Alogp. Finally, the “Scaffold” tab contains all BM scaffolds generated from active molecules of the target, together with the corresponding maximum, mean, median and minimum activity values of the scaffold and the number of underlying compounds. A case study exploring data on the target Cyclin-dependent kinase 5 is provided in the Additional file 1.

Other pages were organized very similarly to the target page, except for specific data. For example, the “Related Molecules” tab on the molecule page contains molecules that have more than 5 active targets in common.

### The graphql query interface

**Fig. 5:**
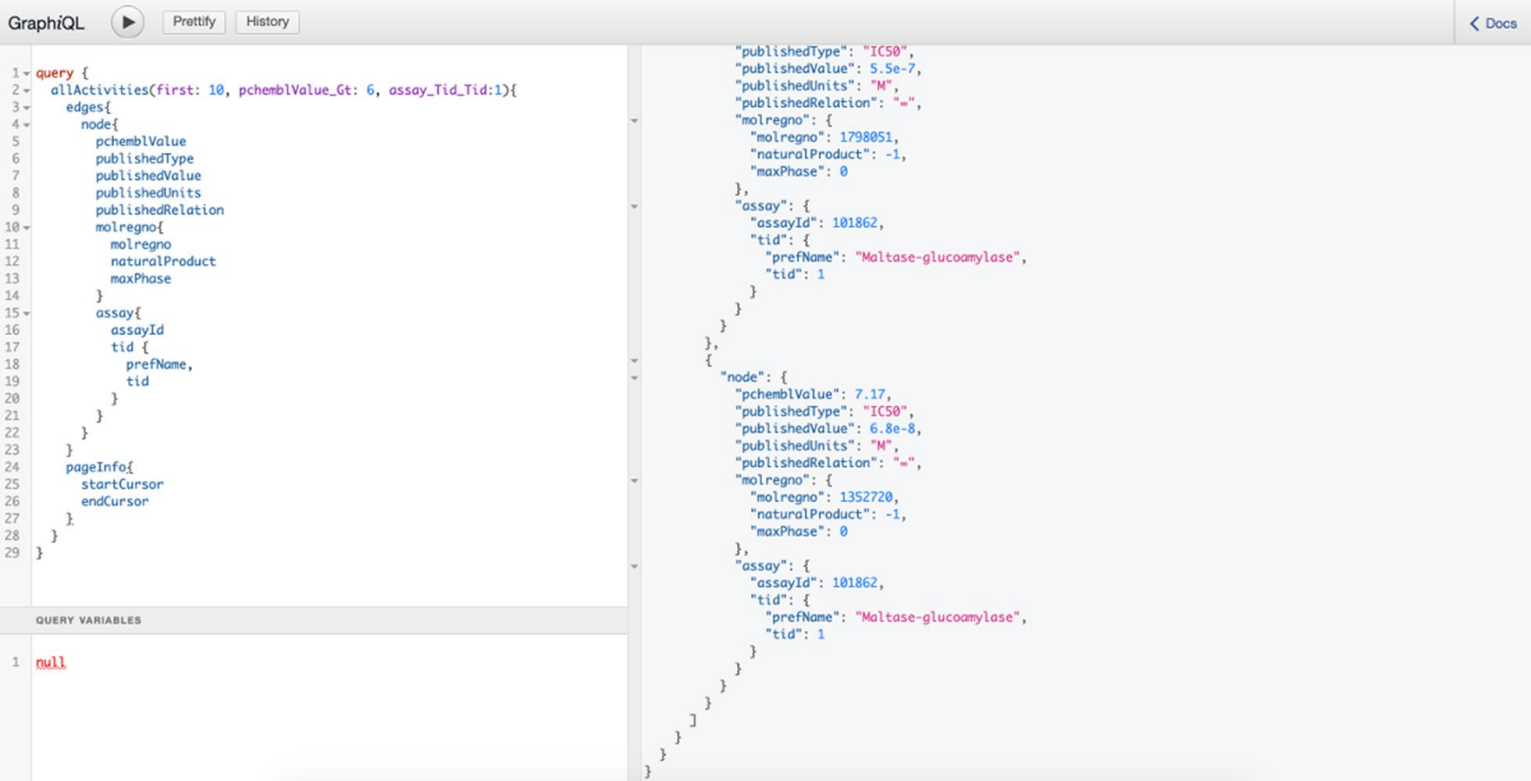
The Graphql query interface (graphiql)

Graphql is a query language for APIs, and gives the user the power to ask for exactly what they need. The graphql query interface (named graphiq) and the documentation are located at http://cadd.pharmacy.nankai.edu.cn/b17r_api/graphql. Figure 5 demonstrates a graphql query (in the left panel) that retrieves the first 10 activities with a pChEMBL value greater than 6 of the target Maltase-glucoamylase (ChEMBL tid 1). The retrieved results are located in the right panel in JSON format.

## Conclusion

We open sourced both the front end and back end of Bioactivity-explorer so that anyone can become directly involved in the development process on GitHub, and researchers who are interested in this tool could build their own local server to use it (which we strongly recommend). In addition to the ChEMBL database, there are still many open and valuable databases that are of great benefit to the drug discovery community; therefore, we expect Bioactivity-explorer to provide an integrated drug development environment for drug discovery and a starting point to integrate more databases.

In summary, based on the ChEMBL database, Bioactivity-explorer consists of 493,430 scaffolds, 31,400,000 MMPs, 1330,220 target–target interactions in terms of shared active compounds, 4526,718 target–target interactions in terms of shared active scaffolds, 97,041,700 molecule–molecule interactions and 14,974 disease-target mappings. For detailed instructions on using Bioactivity-explorer, please refer to the help page.

## Additional file

**Additional file 1.** Case study: Exploring bioactivity data of Cyclin-dependent kinase 5.

## Data Availability

The front end and back end of the tool can be found at https://github.com/jianping-grp/ng-b17r and https://github.com/jianping-grp/b17r, respectively. The tool is also freely available at http://cadd.pharmacy.nankai.edu.cn/b17r.
