# Supplementary material for: Bioactivity-explorer: a web application for interactive visualization and exploration of bioactivity data
Source: J Cheminform. 2019 Jul 10;11:47. doi: 10.1186/s13321-019-0370-7 (PMC6617623; doi:10.1186/s13321-019-0370-7)
Supplement: Supplementary file 1 — Additional file 1. Case study: Exploring bioactivity data of Cyclin-dependent kinase 5. [file 13321_2019_370_MOESM1_ESM.docx]

Case study: Exploring bioactivity data of Cyclin-dependent kinase 5 (ChEMBL4036)

1. Navigate to the target page.

There are three ways to the page of Cyclin-dependent kinase 5 by: (1) inputting target name (Cyclin-dependent kinase 5) or ChEMBL ID (CHEMBL4036), (2) browsing target classification tree and (3) browsing disease classification tree.


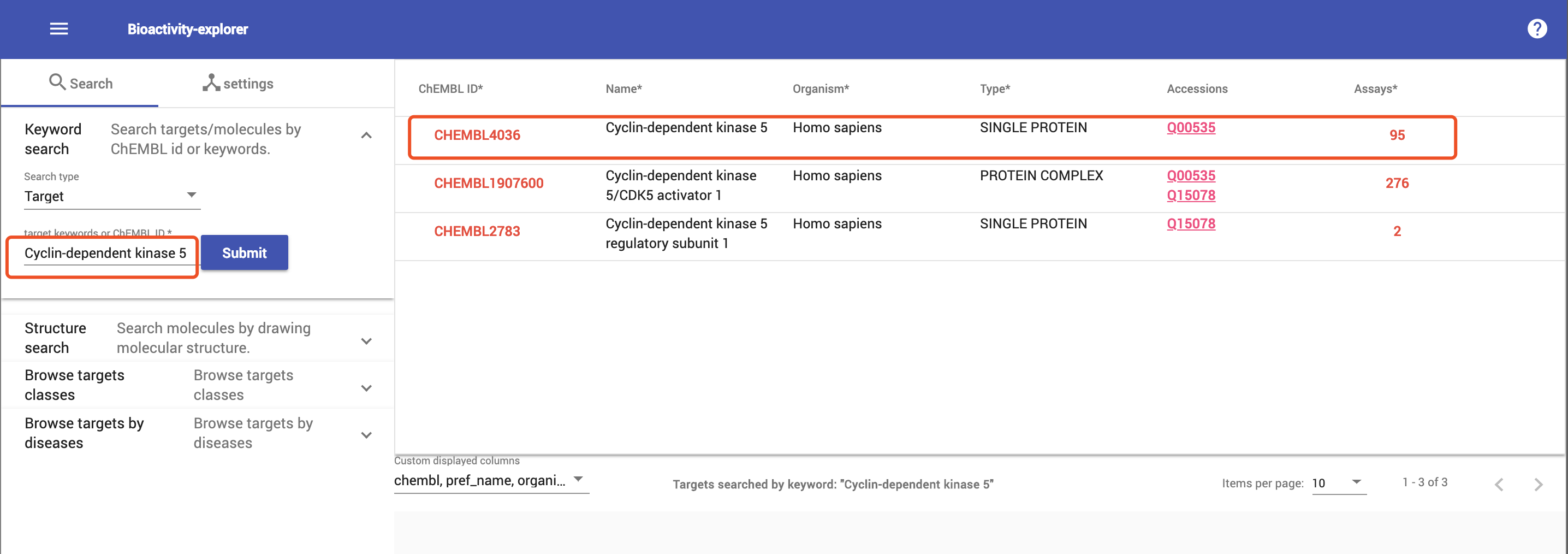


Figure S1. The result interface of search Cyclin-dependent kinase 5 by its name.

1. Target page.

The target page including basic information, activity data table, data statistics charts, MMPs table and molecule scaffolds of the target.


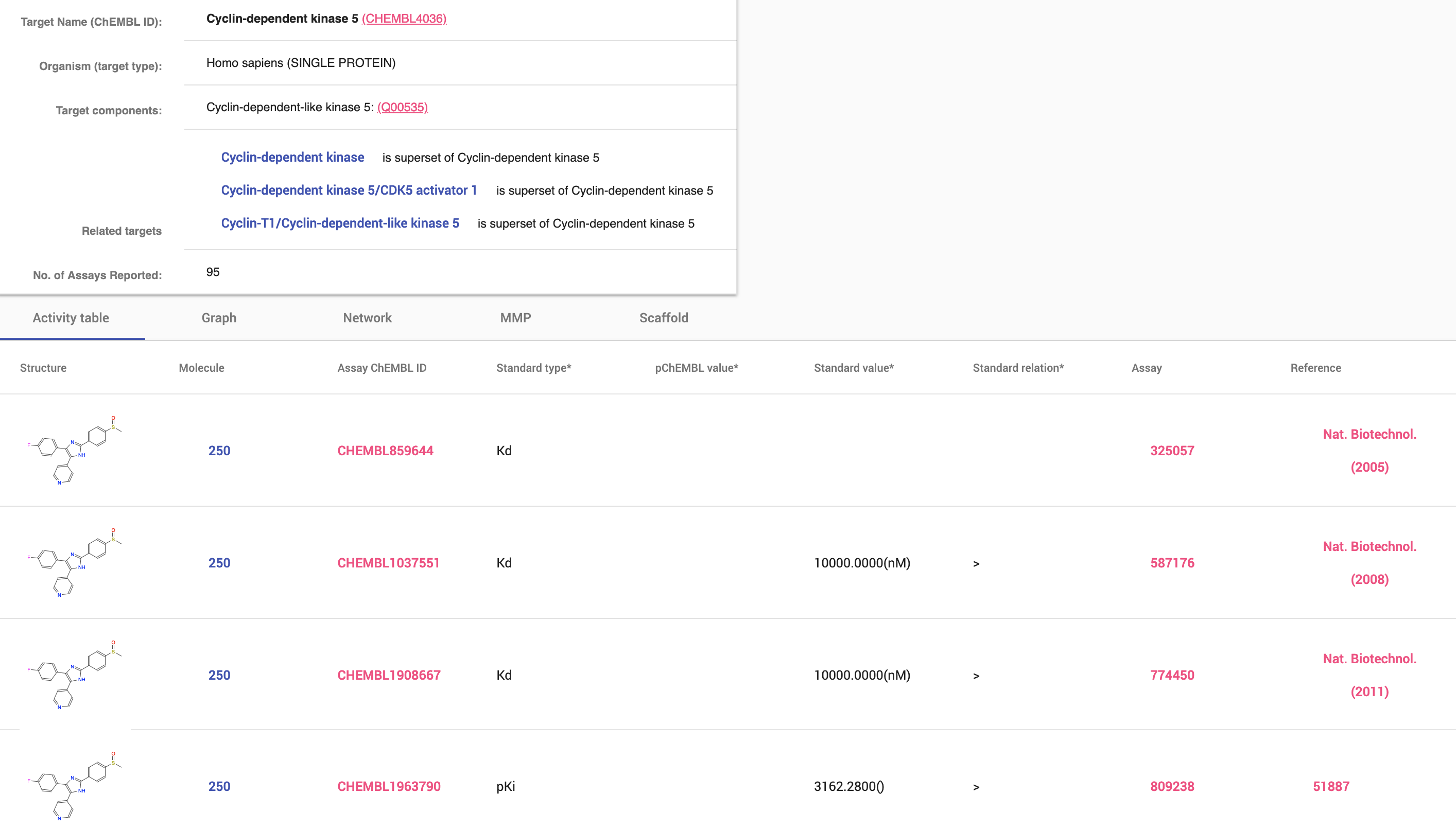


Figure S2. Target page of Cyclin-dependent kinase 5.

1. Bioactivity data statistical visualization

In the “Graph” tab, 5 types of charts were provided for each target. These charts help user to view bioactivities from different perspectives. For example, in the “publication” chart illustrates the research trend of Cyclin-dependent kinase 5, and it became a popular target since 2006. In this year, there were 9 papers about this target published. Double click the node to view corresponding papers. More instruction of using and manipulating the charts in this page can be found on the help page.


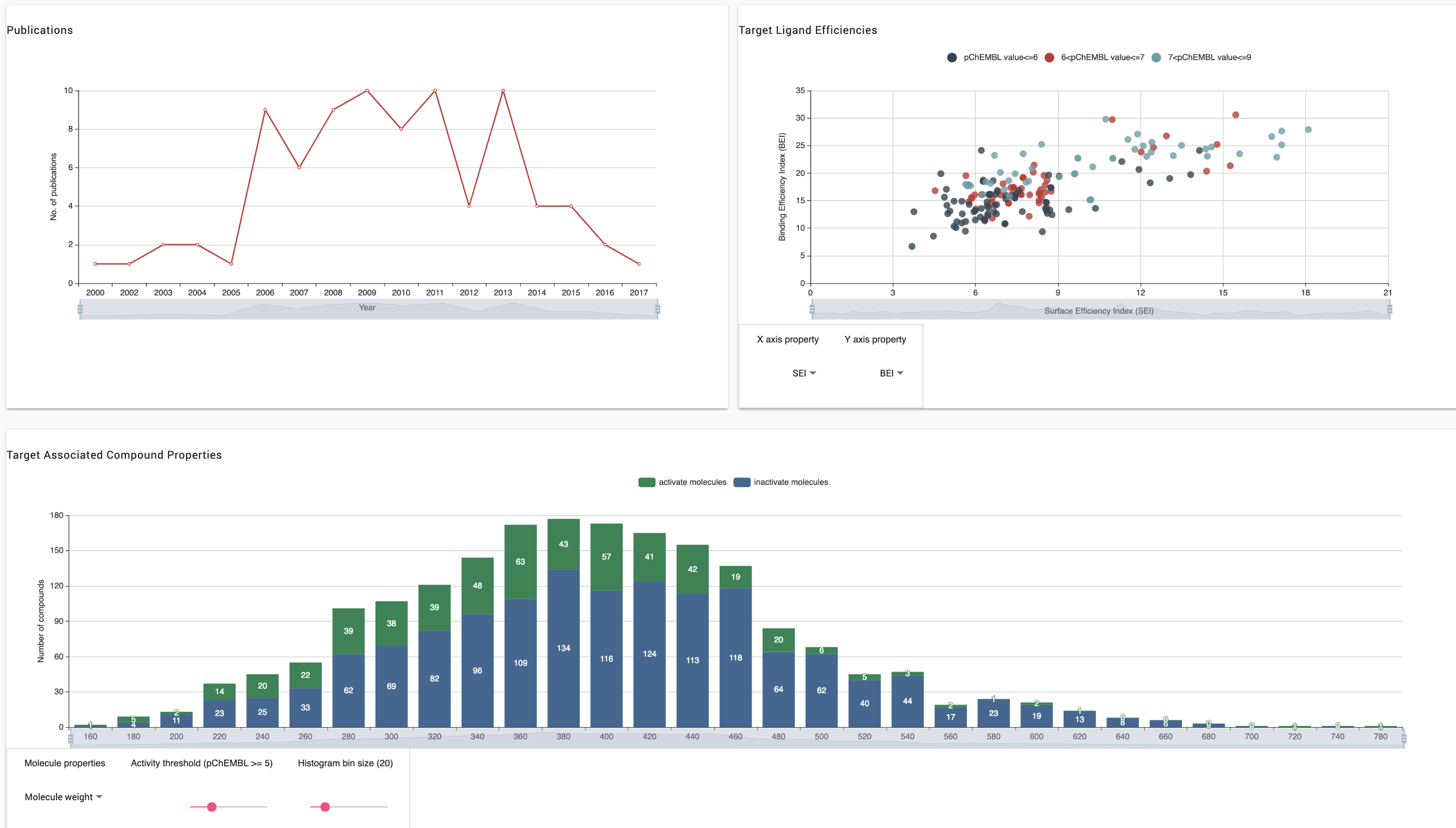


Figure S3. Three kinds of statistical charts of Cyclin-dependent kinase 5.

1. Target interaction network.

Based on the definition that two targets were interacted or related if they have specific number of active molecules in common, a target interaction network (nodes in the network denote targets and edges between two targets denotes the relation of sharing active molecules) was provided in the “Network” tab. Double click a node or a edge between nodes will navigate to a corresponding target page or a shared molecules list page. Options to manipulate the network located at the bottom. For example, nodes can be color by target type or organism. Moreover, the bioactivities underlying the network can be viewed by click the “view” button at the bottom right-hand corner.


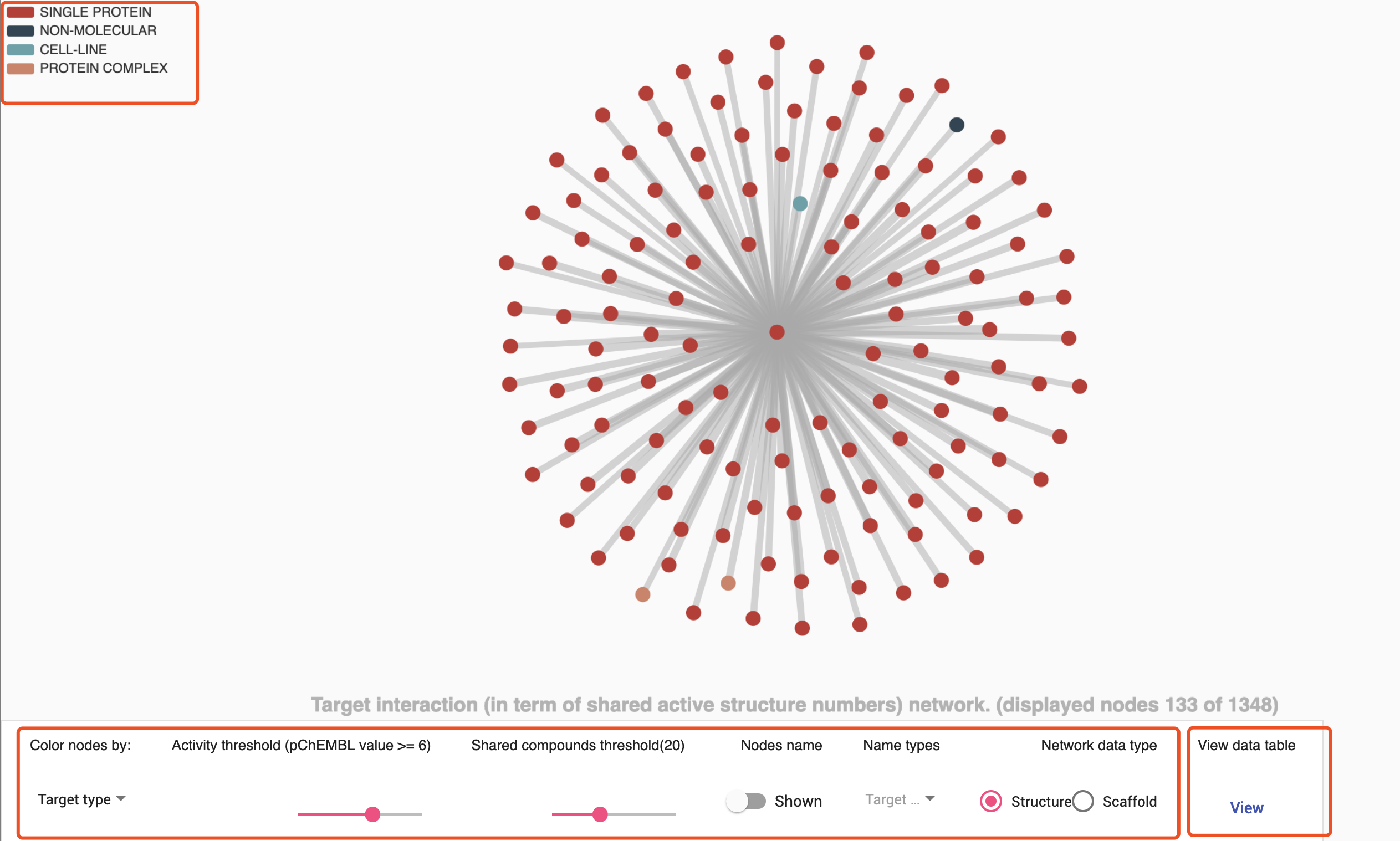


Figure S4. Target interaction network of Cyclin-dependent kinase 5.

1. Matched molecular pairs.

Matched molecular pairs mining from all assays of the target were listed in the “MMP” tab. Beside the activity change of MMPs, properties change, including polar surface area (PSA), number of rotation bonds (RTB), AlogP and molecular weight, can also be viewed by checking the options at lower left-and corner.


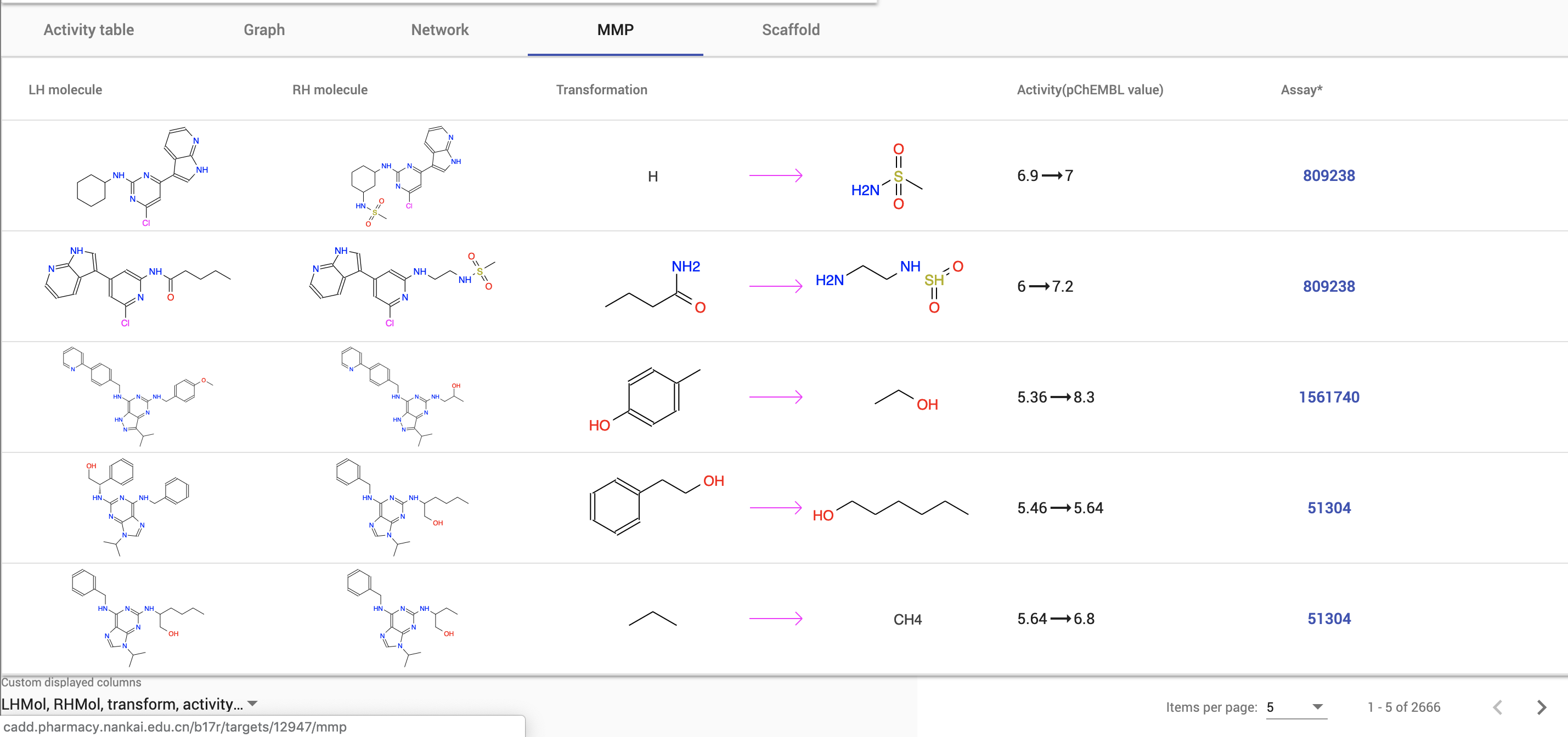


Figure S5. MMPs table of Cyclin-dependent kinase 5.

1. Molecule scaffolds.

At last is the “Scaffold” tab, which providing all BM scaffolds of active molecules of the target. Click the number in the “No. of compound” column to view underlying compounds.


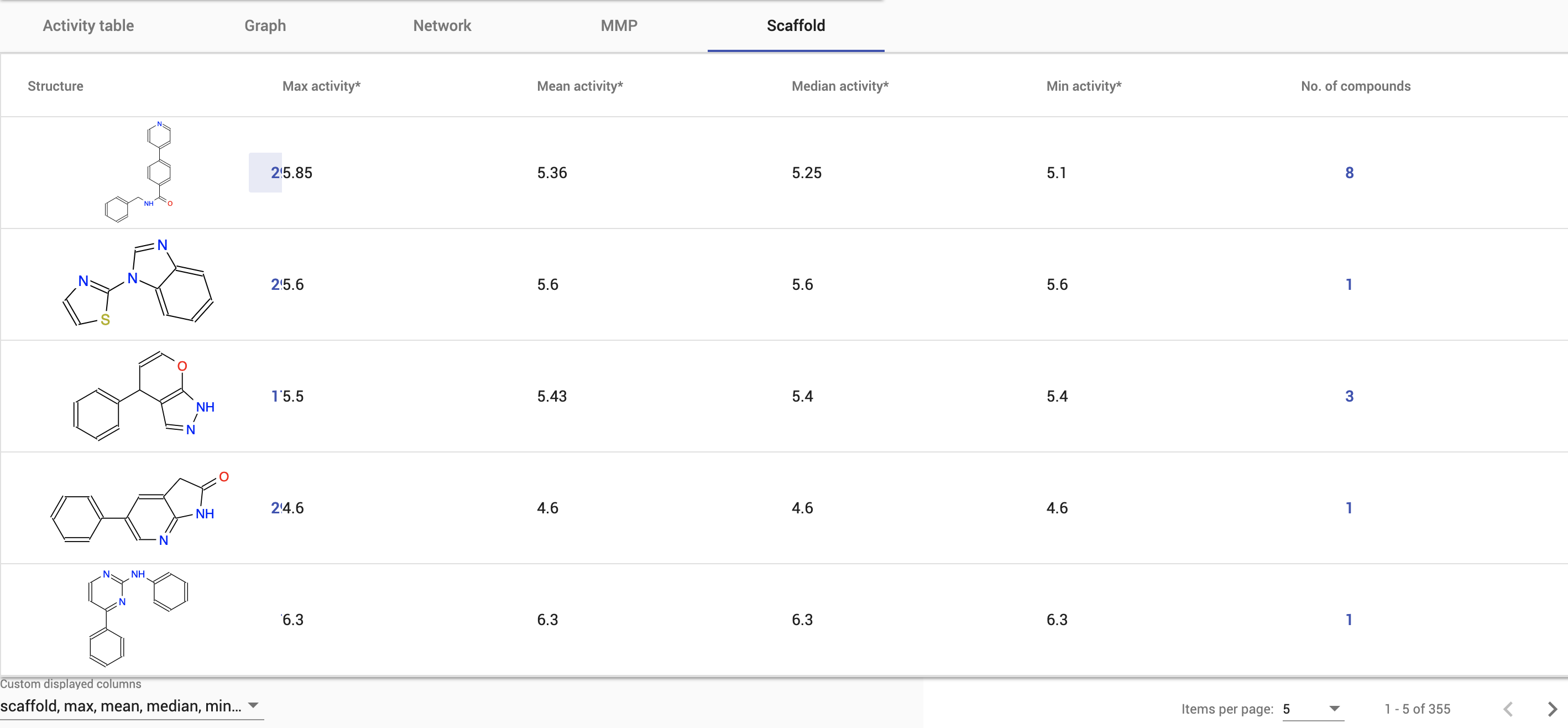


Figure S6. Active molecular scaffolds of Cyclin-dependent kinase 5.
